# The Norwalk Catastrophe

**Published:** 1853-07

**Authors:** 


### THE NORWALK CATASTROPHE.

At a meeting of the delegates to the late Medical Association still remaining in New York, May 12th, the following preamble and resolutions, read by Dr. J. H. Griscom, were adopted:

"*Whereas*, amid the wide-spread affliction caused by the recent catasstrophe at Norwalk, the members of the American Medical Association, recognizing in this mournful event the hand of an all wise Providence, feel called upon to express their grief at the sudden removal from life of Abel L. Pierson, M. D., of Salem, Mass.; Alexander Welch, M. D., of Hartford, Conn.; Josiah Bartlett, M. D, of Stratham, N. H.; Samuel Beach, M. D., of Bridgeport, Conn.; James M. Smith, M. D., and J. H. Gray, M. D., of Springfield, Mass., late members of the Association: And, *whereas*, it is the earnest desire of the members, still present in the City of New York, to record a suitable expression of their feelings upon an occasion equally unprecedented and distressing; therefore,

*Resolved*, That the members have received with profound sorrow the lamentable intelligence of the loss which the community, as well as the profession, have sustained, by the death of so large a number of the American Medical Association.

*Resolved*, That as a suitable, though inadequate, external mark of their grief at the sudden demise of friends from whom they had so recently parted, the members of the Association in general are recommended to wear the usual badge of mourning for the space of thirty days.

*Resolved*, That a Committee of five be appointed to devise some suitable method of commemorating the event, and the worth and professional character of our lamented associates, and recommend their plan at the next meeting of the Association.

*Resolved*, That the members of the Association deeply sympathise with the relatives of the deceased, and that a copy of these resolutions duly authenticated, be transmitted to their respective families."

Drs. Joseph M. Smith, F. C. Stewart, J. W. G. Clements, W. Rockwell, Isaac E. Taylor, E. L. Beadle and John Watson were appointed a committee to consider the best method of providing some memorial in commemoration of the members who perished by the terrible disaster. This committee will report at the meeting of the Association in St. Louis next May.

